# Application Progress of High-Throughput Sequencing in Ocular Diseases

**DOI:** 10.3390/jcm11123485

**Published:** 2022-06-17

**Authors:** Xuejun He, Ningzhi Zhang, Wenye Cao, Yiqiao Xing, Ning Yang

**Affiliations:** 1 Eye Center, Renmin Hospital of Wuhan University, 238 Jiefang Road, Wuhan 430060, China; hexuejun@whu.edu.cn (X.H.); zhangningzhi@whu.edu.cn (N.Z.); caowenye@whu.edu.cn (W.C.); 2 Aier Eye Hospital of Wuhan University, 481 Zhongshan Road, Wuhan 430000, China

**Keywords:** high-throughput sequencing, next-generation sequencing, ocular disease, microRNA, biomarkers

## Abstract

Ocular diseases affect multiple eye parts and can be caused by pathogenic infections, complications of systemic diseases, genetics, environment, and old age. Understanding the etiology and pathogenesis of eye diseases and improving their diagnosis and treatment are critical for preventing any adverse consequences of these diseases. Recently, the advancement of high-throughput sequencing (HTS) technology has paved wide prospects for identifying the pathogenesis, signaling pathways, and biomarkers involved in eye diseases. Due to the advantages of HTS in nucleic acid sequence recognition, HTS has not only identified several normal ocular surface microorganisms but has also discovered many pathogenic bacteria, fungi, parasites, and viruses associated with eye diseases, including rare pathogens that were previously difficult to identify. At present, HTS can directly sequence RNA, which will promote research on the occurrence, development, and underlying mechanism of eye diseases. Although HTS has certain limitations, including low effectiveness, contamination, and high cost, it is still superior to traditional diagnostic methods for its efficient and comprehensive diagnosis of ocular diseases. This review summarizes the progress of the application of HTS in ocular diseases, intending to explore the pathogenesis of eye diseases and improve their diagnosis.

## 1. Introduction

A nucleic acid sequence contains myriad information on the hereditary and evolutionary properties of an organism, which is crucial for improving the diagnosis of a disease. The discovery of the antiparallel double helix structure of DNA by Watson and Crick in 1953 [1] propelled researchers to explore nucleic acid sequences [2,3,4]. In 1975, Sanger et al. published a method for determining DNA sequences [5] using the enzymatic dideoxy DNA sequencing technology [6], which paved the way for modern nucleic acid sequencing techniques.

High-throughput sequencing (HTS), also referred to as next-generation sequencing (NGS) [7], can directly sequence nucleic acids in clinical samples without the traditional culture technology, whose results can then be compared with databases for disease traceability, detection, typing, and drug resistance assessment [8]. The first-generation sequencing technology enables the sequencing of small-molecule DNA fragments; however, the advancement to the second-generation sequencing with improved throughput simplifies the procedure and reduces the cost [7]. The first NGS technology was born in 2000, which opened up new arenas for mammalian genomics research, and sequencing technology has now progressed to the third generation [9]. In addition to the advantages of the previous two generations, third-generation sequencing technology can directly sequence single molecules, such as RNA, without reverse transcription [7]. Commercial NGS technologies are currently used by companies such as Illumina, Oxford, and Pacific Biosciences and involve methods such as 16S rDNA, metagenomic, and single-molecule real-time (SMRT) sequencing. 16S rRNA sequencing mainly studies the species composition, structure, and diversity of a population [10]. Metagenomic sequencing provides a deeper understanding of the characterization of the microbiome complexity, allowing the identification of more species for each sample compared to 16S rRNA sequencing [11]. SMRT is a third-generation sequencing technology that improves the length and accuracy of sequencing compared to the previous sequencing technologies [12]. The emergence of these sequencing technologies with gradually optimized functions has skyrocketed advanced research on molecular biology, and NGS is still improving continuously.

HTS has improved our understanding of diseases and their diagnosis by obtaining genomic information and is widely used to study several clinical problems. For instance, several studies have employed genome sequencing for cancer detection, classification, prognosis prediction, and targeted therapy [13].

Eye diseases can affect visual function to varying degrees, and the etiology of some eye diseases (such as glaucoma) is still unclear. In recent years, the application of HTS in analyzing the intraocular fluid has advanced our understanding of pathogen identification and the pathogenesis of many ocular diseases [14]. Hence, in this article, we review the progress of the application of HTS in ophthalmology and analyze its advantages over traditional diagnostic methods. Furthermore, this review also discusses several HTS-based diagnostic methods to identify new, efficient, and accurate strategies for the diagnosis of eye diseases.

## 2. Traditional Diagnostic Methods to Determine the Cause of Ocular Diseases

### 2.1. Microbial Culture Technology

When several eye diseases occur in a single patient, it is difficult to make a proper etiological diagnosis the first time. Even if the patient has been clinically cured, the etiology remains unclear, which necessitates the identification of the causative agent of infection. The microbiological diagnosis by microbial culture technology has made outstanding contributions to infectious eye diseases, not only by culturing pathogenic microorganisms but also by evaluating their drug sensitivity [15]. It is considered the “gold standard” for diagnosing infectious ocular diseases [16]. The pathogens responsible for eye diseases have diverse characteristics and culture conditions. The culture media used in conventional microbial culture technology can be either aerobic or anaerobic, solid or liquid. The common solid medium includes various types of agar medium, such as brain-heart infusion and soybean-casein digestive agar medium, while the liquid medium includes cooked meat medium [17]. Depending on the localization of the eye disease, the samples used for pathogen culture can be acquired from the conjunctiva, cornea, aqueous humor, or vitreous humor [18,19,20,21]. Gram-positive bacteria account for a large proportion of culture-positive extraocular and intraocular pathogens [20,21]. However, the microbial culture method is limited by the low positive rate of pathogen identification [17]. Therefore, multiple additional methods are required to improve the pathogen detection rate.

### 2.2. Polymerase Chain Reaction (PCR)

Polymerase chain reaction (PCR) is a molecular biology technique that amplifies specific DNA fragments of interest [22]. It consists of three steps: (i) denaturation of template DNA, (ii) annealing (renaturation) between template DNA and primers, and (iii) extension of primers, which subsequently leads to the amplification of the trace amount of starting DNA [23]. A few years after its invention in the 1980s, researchers developed quantitative reverse transcription PCR (RT-qPCR), which can detect and quantify mRNA [24,25].

The examination method employing PCR overcame some of the complications of the microbial culture method, particularly the constraints of time and contamination; therefore, PCR-based methods have become popular in the diagnosis of eye diseases. PCR can assist in detecting acute retinal necrosis and guide initial empirical treatment [26]. Real-time PCR uses peripheral blood mononuclear cell samples to diagnose toxoplasma retinochoroiditis with an extremely high positivity rate [27]. Khanaliha et al. demonstrated that improved real-time PCR using B1 primers is more sensitive than nested PCR for diagnosing toxoplasmosis [27]. Furthermore, Sandhu et al. demonstrated that the diagnostic sensitivity of PCR was 85%, while that of culture was just 17% for endophthalmitis and uveitis [28]. Therefore, PCR is a better method for the etiological diagnosis of ocular diseases. However, PCR has some limitations. Maria et al. found that the positivity rate (27%) of herpes simplex keratitis was low when PCR was used for diagnosis, as it depends on the site of sample acquisition and viral load [29]. The positive detection rate of PCR in blood samples can be as high as 90%, whereas the detection rate in serum samples is negative [27]. Conjunctival swab samples using PCR to detect *Leishmania* DNA in dogs also have low positivity rates (45.45%) [30]. Thus, the inconsistent results obtained by PCR from different sample sources limit its application in diagnosing ophthalmic diseases to a certain extent.

### 2.3. Confocal Microscopy

Confocal microscopy (CM) works on the basic principle that the illumination and detection optics focus on the same diffraction-limited spot, which moves over the target to construct a complete image on the detector [31]. Compared with standard light microscopy, CM reduces the haze of thick and highly scattered samples and can provide optical sections [31]. There are three types of CM: (i) laser scanning in vivo confocal microscope (LS-IVCM), (ii) tandem scanning in vivo confocal microscope (TS-IVCM), and (iii) slit scanning in vivo confocal microscope (SS-IVCM) [32].

Due to its non-invasiveness and high-resolution advantages, CM has been widely used for the etiological diagnosis of ocular diseases. For example, fungal keratitis was rapidly diagnosed by IVCM more than a decade ago [33]. The positivity rate of IVCM for identifying eyelid mite infection pathogens has been shown to reach 100% and can effectively evaluate the function of the meibomian glands [34]. Using CM, Peyman et al. identified cysts and trophozoites of *Acanthamoeba* in the corneas of patients exposed to honey and discovered a new susceptibility factor for *Acanthamoeba* keratitis (AK) [35]. Furthermore, using IVCM, *Acanthamoeba* cysts tested positive in 94.6% of patients diagnosed with AK [36]. Li et al. further demonstrated that *Acanthamoeba* cysts are composed of a low-reflecting light wall and a high-refractive-index nucleus and identified the clinical features that distinguish them from inflammatory cells [36]. Collectively, the above studies illustrate that CM can efficiently detect ocular disease pathogens, especially parasitic infections.

However, IVCM has some limitations. Although it can quickly and accurately identify pathogens, such as fungi and parasites, it cannot distinguish between specific species of pathogenic microorganisms, and there is still some false-positives in the identification of parasitic infections [32,34]. Further, IVCM has been widely used to detect anterior segment lesions [37,38] but has been rarely used to detect posterior segment lesions. Thus, it is critical to make ICVM more sensitive in the future for its widespread use in diagnosing ocular diseases.

## 3. Applications of HTS Technology in Ocular Diseases

### 3.1. HTS Can Identify Ocular Surface Microbes

The ocular surface is directly exposed to air and is rich in microbiota. The microbiota not only maintains the normal microenvironment of the eye but may also harbor potential pathogens causing ocular infectious diseases. Thus, identifying ocular surface microorganisms using HTS is critical for the prevention, diagnosis, and treatment of ocular surface diseases.

#### 3.1.1. Identification of Non-Pathogenic Microorganisms on the Ocular Surface

Huang et al. [39] explored the composition and diversity of bacterial flora in normal conjunctiva using the Illumina HTS. They identified 25 phyla and 526 genera, of which more than ten species of bacteria accounted for more than 76%, including *Corynebacterium* (28.22%), *Pseudomonas* (26.75%), *Staphylococcus* (5.28%), *Acinetobacter* (4.74%), *Streptococcus* (2.85%), *Millisia* (2.16%), *Anaerococcus* (1.86%), *Finegoldia* (1.68%), *Simonsiella* (1.48%), and *Veillonella* (1.00%). Although this study has some differences from the findings of Dong et al. [40], it shows the relevance of HTS in identifying normal conjunctival microbiota (Table 1). Recently, Kuo et al. [41] constructed a model based on a dot hybridization assay (DHA) that combines traditional culture with emerging HTS technologies to study ocular surface microbiota. DHA revealed a higher bacterial bioburden in men than in women, enabled the detection of target pathogens and microbiota, and can monitor ocular surface microbiota for antibiotic resistance [41]. Shivaji et al. [42] were the first to use the NGS technology to explore fungal microbiota on the ocular surface of healthy humans. They detected 65 distinct genera of *Aspergillus*, *Setosphaeria*, and *Malassezia*, among others, with 12–24 genera per microbiome [42] (Table 1). The findings of the above studies show that HTS can comprehensively identify the composition of ocular surface microorganisms, analyze potential pathogenic microorganisms that affect ocular surface homeostasis, and compensate for the shortcomings of traditional detection methods to a certain extent.

#### 3.1.2. Identification of Pathogenic Microorganisms on the Ocular Surface

Fungal keratitis is associated with a high incidence of ocular blindness and has no specific and effective treatment method yet. Zhang et al. [48] used RNA sequencing to find that the expression of the *SPDEF* (SAM pointed domain-containing Ets transcription factor) increased by 154 times on the second day of fungal keratitis in mice compared to the control group. Furthermore, compared with the second day, the expression of the *MARCO* (macrophage receptor with collagenous structure) upregulated approximately 124-fold on the fifth day. SPDEF is a marker of mature goblet cells and is crucial for detecting airway inflammation and colorectal cancer [49,50]. *MARCO* plays a protective role in the body’s resistance to fungal infections [51]. Therefore, the upregulated expression of *SPDEF* and *MARCO* in murine fungal keratitis and their related properties may serve as potential therapeutic targets in fungal keratitis.

Parasites and viruses are common causes of ocular surface infections. To detect *Acanthamoeba*-associated keratitis, Dennis et al. studied NGS-based detection of ribosomal genes in corneal scrapings and reported that NGS could provide information for recognizing *Acanthamoeba* genotypes [52] (Table 1). In addition, the 16S–18S assay can detect potential bacterial and fungal pathogens associated with infectious keratitis. Prajna et al. [43] used an unbiased metagenomic RNA deep sequencing (MDS) to identify conjunctivitis-causing pathogens. The positivity rate of pathogens detected by MDS is as high as 86%, of which more than 71% are human adenoviruses, and approximately 14% are *Vittaforma corneae*. The latter is a rare parasitic microsporidia fungus associated with acute conjunctivitis [43]. Taken together, HTS can identify various microorganisms on the ocular surface, including pathogenic microbes. Moreover, it can differentiate between the genotypes of causative microorganisms, which may aid in the correct diagnosis and treatment of ocular surface diseases.

### 3.2. Application of HTS Technology in Intraocular Diseases

#### 3.2.1. Diabetic Retinopathy (DR)

Diabetic retinopathy (DR) causes severe damage to visual function, accompanied by the formation of several new inflammatory blood vessels. Small RNAs have an important regulatory role in DR [53]. To study the potential role of microRNAs (miRNAs) in proliferative DR (PDR), Chen et al. used NGS to construct a miRNA target gene regulation network, in which the three most influential pathways were Rho protein signal transduction, neurotransmitter uptake, and histone lysine methylation pathways. They also found that the differentiated expression of miRNAs, including miR-150-5p and miR-93-5p, regulated the three pathways [54] (Table 2). Further, pre-miR-150 inhibits neovascularization, whereas miR-93-5p is involved in retinal cell inflammation and apoptosis in PDR [55,56]. Recently, Liu et al. used HTS to demonstrate that the expression of long non-coding RNAs (lncRNAs) was altered in patients with PDR compared to patients with non-proliferative DR, suggesting that lncRNAs may be novel diagnostic and prognostic biomarkers for PDR [57]. Therefore, identifying small RNAs by HTS may help us understand the pathology of PDR.

Other than neovascularization, retinal neurodegeneration and fibrosis are considered manifestations of DR lesions. Through a high-throughput single-cell sequencing analysis, Niu et al. found that overexpression of retinal-binding protein 1 (RLBP1) in Müller glial cells attenuated DR-related neurovascular degeneration in vivo [63]. Dong et al. used RNA sequencing technology to explore changes in gene expression in vascular endothelial cells. They showed that bone morphogenetic protein 4 (BMP4) could significantly promote the expression of SMAD family member 9 (SMAD9), vascular endothelial growth factor (VEGF), and fibrotic factors, suggesting that BMP4 is a potential target for dual-target therapy (anti-VEGF and anti-fibrotic) [64]. These studies demonstrated that HTS provides novel insights into the pathogenic mechanisms of DR-related dysfunction and uncovers potential therapeutic targets for DR treatment.

#### 3.2.2. Uveitis

Uveitis is a common form of eye inflammation. Due to the complications, such as neovascularization and secondary intraocular pressure, uveitis is considered a major cause of eye damage [65]. Thuy et al. found that MDS could efficiently detect abundant pathogens in the intraocular fluid of patients with uveitis. MDS not only identified the RNA virus (rubella virus) that causes uveitis but also implied that the virus may remain in the patient’s eye from the initial infection [44], which could not be detected using PCR. Zhang et al. used HTS to detect vitreous specimens from patients with suspected infectious uveitis and detected various microorganisms, such as varicella-zoster virus, *Candida albicans*, *Propionibacterium acnes*, and *Haemophilus parainfluenzae*, indicating that metagenomic sequencing can be an alternative diagnostic method for uveitis [66]. MDS has limitations in determining the diagnostic threshold: it cannot confirm the survival of the detected microorganisms and is costly. However, it is undeniable that NGS is superior to the other existing diagnostic methods in identifying uveitis-causative pathogens [67].

#### 3.2.3. Endophthalmitis

Infectious endophthalmitis is a rare postoperative complication that might appear after cataract surgery, glaucoma surgery, intravitreal injection, and sometimes after eye trauma [68,69,70]. Although endophthalmitis is rare, its damage to vision is often fatal [71]. Previously, the diagnostic standard for endophthalmitis was microbial culture. However, due to the different culture conditions and methods, as well as the requirements of growing a pathogenic microorganism in the laboratory environment, the positive rate of culture was low (approximately 57.1–70%) [46]. Aaron et al. used deep DNA sequencing to identify viruses (Torque teno virus) and bacteria (*Streptococcus*) in culture-negative samples of patients with suspected endophthalmitis, suggesting the potential of deep-DNA sequencing technology to compensate for the low positive rate of microbial culture and also overcome the limitations of PCR [46] (Table 2). These findings suggest that HTS is superior to the other existing diagnostic methods for identifying rare pathogens.

The pseudorabies virus infection is common in swine, and reports of its invasion in humans are relatively rare. However, Ai et al. recently discovered a case of endophthalmitis caused by the pseudorabies virus using NGS [47]. Although the exact mechanism of the identified viruses in endophthalmitis is yet to be unraveled, HTS complements the comprehensive identification of novel infectious pathogens in humans.

#### 3.2.4. Intraocular Tumor

Intraocular tumors, such as ocular lymphoma and retinoblastoma, occur with relatively insidious symptoms and are diagnosed late, often resulting in permanent vision loss, impacting other systems, and high mortality [72,73]. The clinical presentation of intraocular lymphoma is similar to that of uveitis. The difficulty of biopsy and finding lymphoma cells in the vitreous humor makes it challenging to diagnose the disease [74]. Retinoblastoma often develops at an early age, and when typical symptoms, such as leukocoria and strabismus, appear [75], it often results in enucleation. Early identification of pathogens and mutated genes that cause intraocular tumors is essential for diagnosing intraocular tumors and preventing malignant outcomes. John et al. used MDS to analyze the aqueous humor of patients with intraocular lymphoma. They detected Epstein-Barr virus and human herpesvirus RNAs in the aqueous humor of one patient (Table 1). Another patient had an uncommon mutation in the *MYD88* associated with B-cell lymphoma [45], which might be used as a marker in the diagnosis and treatment of intraocular lymphoma. Retinoblastoma is believed to be caused by mutations in *RB1* and *MYCN* genes [76]. However, the poor prognosis of the disease may also be related to other factors. In a study of retinoblastoma using NGS, Francis et al. found that mutations in *BCOR* are related to the poor prognosis of retinoblastoma [77]. Thus, the application of HTS in intraocular tumors has not only improved the diagnosis but also deepened our understanding of the underlying mechanisms of the development of intraocular tumors.

#### 3.2.5. Glaucoma

Glaucoma is an ocular disease characterized by the loss of retinal ganglion cells and thinning of the retinal nerve fiber layer, causing irreversible loss of vision [78]. The exact pathological mechanisms underlying glaucoma remain unclear. It is believed that the pathological process of glaucoma is related to intraocular pressure, age, and genetic factors [60]. Thus, exploring the factors influencing glaucoma, including miRNAs, is critical. miRNAs are pivotal for the post-transcriptional regulation of gene expression, involving processes such as cell differentiation, growth, and death [79].

Liu et al. used NGS to sequence miRNAs in the aqueous humor of patients with primary open-angle glaucoma (POAG) with different degrees of visual field damage. They identified 16 differentially expressed miRNAs, including hsa-miR-205-5p and hsa-miR-206 [58]. Liu et al. further identified the circulating hsa-miR-210-3p as a potential diagnostic marker for severe POAG [59]. In addition, thiamine and purine metabolism pathways related to the differential expression of miRNAs may play a role in the occurrence and development of optic neuropathy in glaucoma [58]. After conducting small-molecule RNA sequencing of aqueous humor and plasma of patients with POAG, Hubens et al. found that there was no differential miRNA expression in plasma, but there were four upregulated miRNAs (hsa-miR-30a-3p, hsa-miR-143-3p, hsa-miR-211-5p, and hsa-miR221-3p) and three downregulated miRNAs (hsa-miR-92a-3p, hsa-miR-451a, and hsa-miR-486-5p) in the aqueous humor [60]. The study suggested that hsa-miR-221-3p and hsa-miR-143-3p, the miRNAs upregulated in the aqueous humor, are potential biomarkers for diagnosing glaucoma [60]. In a study of miRNAs in the aqueous humor of patients with normal-tension glaucoma, Seong et al. identified eight differentially expressed miRNAs, including hsa-let-7c-5p and hsa-miR-375, associated with apoptosis, autophagy, and neurodegeneration [61] (Table 2). Thus, understanding the differential expression of miRNAs and the regulation of related pathways in glaucoma will pave the way for unraveling the mechanism of its occurrence and progression.

### 3.3. Application of HTS in the Refractive System

Myopia is a common eye disease that causes vision impairment. It can often be corrected by wearing glasses and refractive surgery [80]. However, structural changes in the eye due to myopia are irreversible, leading to an increased risk of retinal detachment and other serious vision-impairing conditions [81]. To date, the pathological mechanisms underlying myopia remain unclear. Many studies have explored the mechanisms of myopic lesions. Chen et al. demonstrated that exosomal miRNAs might be related to the occurrence of myopia [82]. Edita et al. also showed that the expression of some miRNAs was upregulated in the blood of patients with myopia [83]. Zhu et al. used NGS to analyze the aqueous humor in patients with high myopia and found 17 differentially expressed miRNAs, including hsa-let-7i-5p, hsa-miR-127-3p, and hsa-miR-98-5p [62] (Table 2). The differentially expressed miRNAs were thought to be involved in the pathology of myopia through the TNF, MAPK, PI3K-Akt, and HIF-1 signaling pathways [62]. Although the role of each miRNA in the development of myopia requires further verification, HTS helped identify miRNAs that might play a pivotal role in this disease.

## 4. Discussion

### 4.1. Advantages and Limitations of Traditional Pathogen Identification Techniques

It is difficult to determine the best method for detecting ocular pathogenic microorganisms. Traditional culture techniques can successfully isolate various microorganisms, such as bacteria and fungi (Table 1), and positive culture results can help make a clear etiological diagnosis of the corresponding disease. However, the culture technique can only isolate specific microorganisms that meet the conditions of the Petri dish, which fails to show the overall composition of the microbial community (Table 3). Moreover, culture techniques typically require several weeks or more to provide conclusive results, and the rate of positive results is low [15], requiring the aid of other pathogen detection methods.

PCR can use a small amount of nucleic acid to identify the pathogen by detecting the amplified sequence. Previously, quantifying the amount of PCR product was extremely difficult, and real-time PCR overcame this limitation by introducing fluorescent dyes or probes into the reaction. However, in PCR experiments, only predetermined sequences can be distinguished, resulting in many potential pathogens not being identified [27,29]. The final PCR results vary due to the different sources of samples, and there may be room for further improvement in its sensitivity (Table 3).

Due to its non-invasive characteristics, CM can assist in the quick and repeated examination of eye diseases caused by certain microbial infections, effective diagnosis, and follow-up observations during treatment. However, the efficacy of CM is also limited since it cannot discriminate between specific types of pathogens.

### 4.2. Application Status and Prospects of HTS Technology

With the advancement of HTS technology, we are gaining increasing information on genes, transcripts, and non-coding RNAs (such as miRNA) [43,59,62]. The recent full-length nanopore 16S sequencing technology has the advantages of portability, low cost, and rapid sequencing [84]. It is increasingly used in the etiological diagnosis of various eye diseases and has also contributed greatly to exploring the normal microbiota that maintains ocular surface homeostasis [39,42]. HTS can accurately diagnose rare pathogens that traditional testing methods cannot detect, identify causative factors that may have been overlooked [17], and compensate for the shortcomings of traditional methods. Furthermore, it can recognize specific nucleic acid sequences, detect genetic variants that may be closely related to ocular tumorigenesis [76], and identify differentially expressed miRNAs in ocular lesions, such as DR and glaucoma [54,58]. The technical advantages of HTS have opened up new avenues for studying the pathogenic mechanisms of various clinical diseases and provide new directions for follow-up treatment. Thus, HTS has great future applications in the field of ophthalmology.

However, to efficiently utilize HTS in ophthalmology, many of its limitations need to be resolved (Table 3). MDS can theoretically detect all pathogens in clinical samples. However, it is challenging to distinguish whether a microbe is a contaminant from a laboratory or reagent or is it the actual causative agent of the disease [43]. Huang et al. and Dong et al. used HTS to analyze the conjunctival flora on the ocular surface of healthy humans [39,40]. Although the results of the two studies had some overlap, there were also large differences (Table 1). Furthermore, Liu et al. [58] and Hubens et al. [60] performed HTS on differentially expressed miRNAs in patients with POAG, and their results were also dissimilar (Table 2). These dissimilarities reported in the above studies investigating the same ocular disease using aqueous humor raise several questions and concerns. Firstly, why did different HTSs give inconsistent results with the same sample type? Secondly, how can consistency in multiple HTS be achieved? Thirdly, will there be a bias due to individual patient differences even in the same sample type? Finally, are there any contamination issues with the obtained samples?

Nevertheless, there are some objective reasons for the current predicament faced by HTS. HTS technology is improving and progressing gradually with time, which may be a reasonable explanation for the differences in the results obtained using the same method at different times. In addition, clinical samples are mostly obtained during cataract surgery, and it is difficult to obtain samples such as aqueous humor from healthy individuals. Whether these factors interfere with the HTS results needs to be further explored.

## 5. Conclusions

This review provides an overview of the current progress in the application of HTS in ocular diseases and compares it with several traditional ocular pathogen detection methods. The ability of HTS to determine nucleic acid sequences has unparalleled advantages in the detection of genes, transcripts, and non-coding RNAs. In ophthalmology, HTS can identify not only normal flora but also various pathogenic microorganisms that cause eye diseases. HTS has helped discover rare pathogens and differentially expressed miRNAs involved in ocular diseases. Some of the differentially expressed miRNAs can be used as biomarkers, which will help us elucidate the pathogenesis of the corresponding eye disease. The advantages of nucleic acid sequence recognition and high throughput allow HTS to recognize thousands of nucleic acid sequences simultaneously, covering almost all types of microorganisms in the sample. However, the exact pathogen cannot be specifically identified the first time. In addition, HTS still has some shortcomings, such as being slightly expensive and time-consuming, but in general, the contribution of HTS in the application of eye diseases is indelible. In the future, we believe that HTS will greatly contribute to the understanding of the pathogenic mechanisms of eye diseases and their prevention, diagnosis, and treatment.

## Figures and Tables

**Table 1:** Ocular microbes identified using high-throughput sequencing.

Term	Bacteria	Fungal	Virus	Parasite	Sample	References
MiSeq Illumina Sequencing Platform	*Corynebacterium, Pseudomonas, Staphylococcus, Acinetobacter, Streptococcus, Millisia, Anaerococcus, Finegoldia, Simonsiella, Veillonella*	*Aspergillus, Setosphaeria, Malassezia, Haematonectria*	—	—	Conjunctival swab samples	[39,42]
GS-FLX 454	*Pseudomonas, Propionibacterium, Bradyrhizobium, Corynebacterium, Acinetobacter, Brevundimonas, Staphylococci, Aquabacterium, Sphingomonas, Streptococcus, Streptophyta* *,* *Methylobacterium*	—	—	—	Conjunctival swab samples	[40]
Metagenomic deep sequencing	—	Cryptococcus neoformans	Human adenovirus, Herpes simplex virus, Rubella virus, Epstein-Barr virus, Human herpesvirus 8	*Vittaforma corneae, Toxoplasma gondii*	Conjunctival swab samples, Intraocular fluid samples, Aqueous fluid	[43,44,45]
Illumina HiSeq 750	*Staphylococcus, Streptococcus*	—	Torque Teno Virus	—	Aqueous fluid, Vitreous samples	[46]
Next-generation sequencing	*Thermoanaerobacter wiegelii, Corynebacterium urealyticum, Haloquadratum walsbyi, Brachyspira pilosicoli, Candidatus Nitrososphaera*	*Cryptococcus gattii*	Pseudorabies virus, Suid herpesvirus 1, Bovine herpesvirus 5	—	Vitreous humor	[47]

**Table 2:** Differentially expressed miRNAs in intraocular diseases identified by high-throughput sequencing.

Term	miRNA	Ocular Disease	Sample	References
HiSeq4000 platform	↑: hsa-miR-99b-5p ↓: miR4433b-3p, hsa-miR-150-5p, hsa-miR-30c-5p, hsa-miR-16-2-3p, hsa-miR-1827, hsa-miR-140-3p, hsa-miR-93-5p	PDR	Aqueous humor	[54]
HiSeq4000 platform	↑: hsa-miR-205-5p, hsa-miR-206, hsa-miR-16-5p, hsa-miR-501-3p, hsa-miR-409-3p, hsa-miR-200a-3p, hsa-miR-200b-3p, hsa-miR-382-5p, hsa-miR-543, hsa-miR-136-3p, hsa-miR-30c-2-3p, hsa-miR-139-5p, hsa-miR-340-5p, hsa-miR-488-3p, hsa-miR-202-5p, hsa-miR-369-5p	POAG	Aqueous humor	[58]
HiSeq4000 platform	↑: hsa-miR-885-5p, hsa-miR-210-3p, hsa-miR-3149	POAG	Aqueous humor	[59]
Illumina NovaSeq 6000	↑: Hsa-miR-30a-3p, hsa-miR-143-3p, hsa-miR-211-5p, hsa-miR221-3p ↓: hsa-miR-92a-3p, hsa-miR-451a, hsa-miR-486-5p	POAG	Aqueous humor	[60]
NextSeq500 system	↑: hsa-let-7a-5p, hsa-let-7c-5p, hsa-let-7f-5p, hsa-miR-192-5p, hsa-miR-10a-5p, hsa-miR-10b-5p, hsa-miR-375, and hsa-miR-143-3p	NTG	Aqueous humor	[61]
Illumina HiSeq4000 sequencing platform	↑: miR-29b, let7b/c/e, miR-214, miR-103, miR-98	High myopia	Aqueous humor	[62]

↑, upregulated; ↓, downregulated; hsa, Homo sapiens species; miR, microRNA; PDR, proliferative diabetic retinopathy; POAG, primary open-angle glaucoma; NTG: normal-tension glaucoma.

**Table 3:** Comparison of methods to identify the etiology of eye disease.

Method	Advantages	Limitations	References
Microbial Culture	High specificity.	Time-consuming; low positivity rate.	[15,19,41]
Polymerase Chain Reaction	Samples can be expanded indefinitely; Diagnosis at the molecular level.	Sample site dependence; Only predetermined sequences.	[27,28,29,30]
Confocal Microscopy	Non-invasive; Quick diagnosis; Can be repeated many times.	Inability to type microbes; Limitation of available parts	[34,35,36] [37,38]
High-throughput Sequencing	High positive rate; High sensitivity; Can detect RNA directly; Diagnosis at the molecular level.	Expensive; Low specificity.	[42,48,52] [43,44]

## Data Availability

Not applicable.
